# ABO Blood Group in Relation to COVID-19 Susceptibility and Clinical Outcomes: A Retrospective Observational Study in the United Arab Emirates

**DOI:** 10.3390/life12081157

**Published:** 2022-07-29

**Authors:** Wael Hafez, Shougyat Ahmed, Nihad Abbas, Kamran Ahmed, Samera Kamran, Arun Arya, Srinivasa Raghu Rao, Mahmoud Abdelshakor, Sara Ali, Honeymol Sebastian, Mishal Tariq, Kumar Lal, Ahmed Abdelrahman

**Affiliations:** 1 NMC Royal Hospital, Abu Dhabi P.O. Box 35233, United Arab Emirates; shougyat.ahmed@nmc.ae (S.A.); nihad.abbas@nmc.ae (N.A.); kamran.ahmed@nmc.ae (K.A.); samera.kamran@nmc.ae (S.K.); arun.arya@nmc.ae (A.A.); srinivasa.rao@nmc.ae (S.R.R.); mohamed.mahmoud@nmc.ae (M.A.); sara.ali@nmc.ae (S.A.); honeymol.sebastian@nmc.ae (H.S.); mishal.tariq@nmc.ae (M.T.); kumar.lal@nmc.ae (K.L.); ahmed.abdelrahman@nmc.ae (A.A.); 2 The Medical Research Division, Department of Internal Medicine, The National Research Center, Cairo 12622, Egypt; 3 Internal Medicine Department, Zagazig School of Medicine, Zagazig 44519, Egypt

**Keywords:** ABO blood group, COVID-19, severity, pneumonia, viral clearance, UAE

## Abstract

(1) Background: The association between ABO blood groups and COVID-19 outcomes was investigated in several studies. The results were controversial. This study aimed to explore the association between ABO blood groups and COVID-19 outcomes. (2) Methods: This retrospective study included 303 COVID-19 patients treated at the NMC Royal Hospital in the United Arab Emirates between 8 April 2020 and 30 June 2020. (3) Results: The mean age of patients included in the study was 39.3 ± 10.7 years, and 72.9% of patients were males. The prevalence of blood groups O, A, B, and AB was 40.3%, 27.7%, 25.1%, and 6.9%, respectively. The correlation between ABO blood groups and COVID-19 outcomes was insignificant except in the AB group, with significantly higher odds of disease severity. Increased age, higher body mass index (BMI), and being of male gender increased the risk for pneumonia among all blood groups. Both increased age and higher BMI increased the risk of mortality, and increased age increased the risk of disease severity. Troponin and platelet counts were significantly different in the A group compared to the non-A groups. Time to viral clearance was not different among blood groups. However, adjustment for Rh groups resulted in a significantly shorter time in the B group. (4) Conclusions: There was no significant association between ABO blood groups and COVID-19 outcomes, with the exception of group AB.

## 1. Introduction

The severe acute respiratory syndrome coronavirus 2 (SARS-CoV-2) is a current global health crisis that has emerged and spread worldwide [1,2]. As of 11 July 2022, around 560,889,536 coronavirus disease 2019 (COVID-19) cases and 63,734,477 deaths have been reported [3]. While some SARS-CoV-2 infections cause no symptoms, the majority of patients experience moderate symptoms, including fever, dry cough, and shortness of breath [4]. The condition may progress to pneumonia, hypoxemia, severe respiratory distress, and multiorgan failure leading to death [1].

According to some clinical observations, several demographic characteristics, including age, gender, and certain chronic medical conditions, appear to be risk factors for becoming infected with SARS-CoV-2 and developing serious outcomes [5]. No physiological biomarker can accurately predict COVID-19 susceptibility, but viral infection susceptibility has previously been linked to ABO blood groups. ABO blood groups are carbohydrate epitopes on the outer surface of human cells. The trisaccharide moieties GalNAc1-3- (Fuc1,2)-Gal-, and Gal1-3- (Fuc1,2)-Gal- are the antigenic determinants for A and B blood groups, respectively, whereas Fuc1,2-Gal- is the antigenic determinant for the O blood group. While blood types are inherited genetically, environmental influences may impact which group is inherited to the next generation more frequently than others [6,7]. It was also found that people with blood group O were less susceptible to SARS-CoV-2 infection [8].

The results regarding the role of ABO blood groups in the pathogenesis of COVID-19 are controversial. Primary studies showed no association between ABO blood groups and SARS-CoV-2 infection [9]. However, recent studies reported that blood group O is more linked to a reduced risk of COVID-19 than non-O blood groups. In contrast, blood group A is linked to higher odds of SARS-CoV-2 infection and severe outcomes [10,11,12]; similar findings were observed regarding the risk of cardiovascular complications among patients with blood group O versus patients with blood group A [13].

The biologic mechanisms underlying these observations could be related to the ABO group in general (e.g., the formation of neutralizing antibodies against protein-linked N-glycans) or to other biologic consequences of the discovered variant, such as von Willebrand factor (VWF) stability [14]. The presence of a galactose compound as an end group saccharide in the blood group is one potential mechanism involved in the elevated risk. A galactose amine could be found in this position in blood types B and O, which may explain their differences. In addition, it was shown that the SARS-spike COV-2 protein could bind to carbohydrates. Furthermore, viral entry could be facilitated by the presence of significant affinity between the A antigen and the virus [15].

Our study aimed to investigate the association between the ABO blood group and the outcomes of COVID-19, including the risk of infection, clinical presentation, and disease severity among hospitalized patients.

## 2. Materials and Methods

### 2.1. Study Design and Participants

This was a non-interventional, observational retrospective analysis of the medical records of 303 adult COVID-19 patients aged 18 years or older who were treated at NMC Royal Hospital, Khalifa City, Abu Dhabi, the United Arab Emirates (UAE), between 8 April 2020 and 30 June 2020. Patients were evaluated for SARS-CoV-2 infection using reverse transcriptase–polymerase chain reaction (RT-PCR) assay using specimens from nasopharyngeal swabs under complete aseptic conditions at the time of presentation or just a few days earlier. An ABO blood group test was also performed.

The identifiable patients’ information was not used, and patients’ confidentiality was maintained throughout the study. Storage of the data was in accordance with the NMC MRD (Medical Records Department) policy.

The study was conducted following good clinical practice. Investigators and clinical research team members are qualified by training and experience. The study was reviewed and approved by the NMC Central Scientific Committee (NMCHC/CR/AUH/CSC/APP/002), the NMC Regional Research Ethics Committee (NMCHC/CR/AUH/APP/002), and the Department of Health, Abu Dhabi, the UAE Committee (DOH/CVDC/2020/2358).

### 2.2. Data Collection

Clinical and demographic data from current and prior clinical encounters were collected from the Insta hospital management system (HMS), a cloud-based electronic health record (EHR). Other extracted data included ethnicity, gender, age, coexisting diseases, clinical presentation, severity grade, peripheral oxygen saturation, disease outcomes, ABO blood group, and inflammatory markers indicative of severe infection (platelet and lymphocyte count, C-reactive protein [CRP], ferritin, lactate dehydrogenase [LDH], fibrinogen, and D-dimer). All patients had either a chest X-ray and/or a chest CT on presentation. Some had follow-up chest X-rays and/or chest CTs at different intervals according to clinical progression.

### 2.3. ABO Blood Grouping and Rh Genotyping

ABO blood grouping and Rh genotyping were examined with the help of the hemagglutination technique using the Ortho BioVue System ABO-Rh Grouping Cassette, consisting of glass beads and reagent contained in the column. Following the addition of the red blood cells and subsequent centrifugation of the cassette, agglutinated red blood cells were caught by the glass beads, while non-agglutinated red blood cells travelled to the bottom of the column [16].

### 2.4. Statistical Analysis

A descriptive analysis of demographic, clinical, laboratory and disease-related variables was provided for all cases (mean, standard deviation (SD), median, and number (n), %). Univariate analysis was used to investigate the correlation between ABO blood groups and disease severity and mortality, assessed using a Chi-square test. Logistic regression models were performed to investigate the association between blood groups and COVID-19 outcomes (making “O group” and “A group” the reference groups). Comparative analysis was performed to compare laboratory findings between all blood groups among inpatients. Linear regression models were also used to investigate the association between blood groups and laboratory findings among COVID-19 inpatients (making “O group” and “A group” the exposed groups). The Kaplan–Meier curve and log-rank test were performed to analyze time to viral clearance among all blood groups. Multivariate Cox proportional hazard models were used to explore the association between viral clearance rate (rate ratio) and blood groups. All analysis was performed using statistical analysis using R Software version 3.5.2 (20 December 2018)—“Eggshell Igloo”. Copyright (C) 2018 The R Foundation for Statistical Computing, Platform: i386-w64-mingw32/i386 (32-bit) “source”, Egypt. A two-sided *p*-value < 0.05 was considered statistically significant.

## 3. Results

### 3.1. Demographic and Clinical Characteristics of Patients

The study was carried out on 303 COVID-19 patients; blood group O was more prevalent, representing 40.3% of patients, compared to A (27.7%), B (25.1%), and AB (6.9%). The mean age was 38.6 ± 11.3 years for group O, 38.4 ± 10.3 years for group A, 41.9 ± 10.8 years for group AB, and 40.7 ± 9.9 years for group B. BMI was calculated according to the formula weight (kg)/height (m)^2^ so the mean BMI was 27.4 ± 5.0, 26.6 ± 5.7, 26.8 ± 3.3, and 27.2 ± 3.8 for groups O, A, AB, and B, respectively.

Males represented 68.0%, 69.0%, 81.0%, and 82.9% while females represented 32.0%, 31.0%, 19.0%, and 17.1% from groups O, A, AB, and B, respectively, showing a non-statistically significant difference (*p* = 0.085). Regarding race, Asians represented 86.9%, 83.3%, 85.7%, and 92.1% from groups O, A, AB, and B, respectively, showing a non-statistically significant difference (*p* = 0.251). Positive RH patients represented 83.9%, 93.5%, 95.2%, and 94.2% from groups O, A, AB, and B, respectively, also without a statistically significant difference (*p* = 0.077).

Co-morbidities investigated in these cases were hypertension, diabetes mellitus, and cardiovascular diseases, and these did not show any statistically significant difference between all blood groups. Hypertension represented 8.4%, 5.6%, 5.0%, and 10.6%, while diabetes represented 6.0%, 14.9%, 9.5% and 10.6% and cardiovascular diseases represented 1.7%, 2.4%, 0.0% and 5.3% from groups O, A, AB, and B, respectively.

The univariate comparative analysis also showed a non-significant association between different blood groups and different outcomes of COVID-19, including the development of pneumonia, intensive care unit (ICU) admission, severe disease, the need for oxygen, and mortality (Table 1).

The logistic regression model showed that the odds of disease severity among patients within the AB blood group increased approximately three-fold when using blood group O as a reference blood group (OR = 3.09, 95%CI: 0.97–9.05, *p* = 0.044) (Table 2).

In contrast, the remaining odds of pneumonia, ICU admission, mortality, and disease severity did not show significant association with the patients’ blood groups, nor when compared to blood group O and blood group A (Table 2 and Table 3).

Furthermore, an adjustment for certain patients’ demographics showed that for each one-year increase in a patient’s age, the odds of pneumonia, severity, and mortality significantly increased by about 10% (OR = 1.10, 95%CI: 1.06–1.13, *p* < 0.001), 10% (OR = 1.10, 95%CI: 1.06–1.14, *p* < 0.001), and 16% (OR = 1.16, 95%CI: 1.08–1.26, *p* < 0.001), respectively, in all patients’ blood groups, taking into account patients’ BMI and sex. The results also showed that the odds of pneumonia and severe disease significantly increased by about 14% (OR = 1.14, 95%CI: 1.07–1.23, *p* < 0.001) and 10% (OR = 1.10, 95%CI: 1.03–1.19, *p* = 0.009), respectively, for each one-unit increase in a patient’s BMI in all patients’ blood groups considering patients’ age and sex. Additionally, the odds of pneumonia in males increased approximately three-fold compared with females in all patient blood groups, considering the patients’ age and BMI (OR = 3.05, 95%CI: 1.59–5.99, *p* = 0.001).

**Figure 1:**
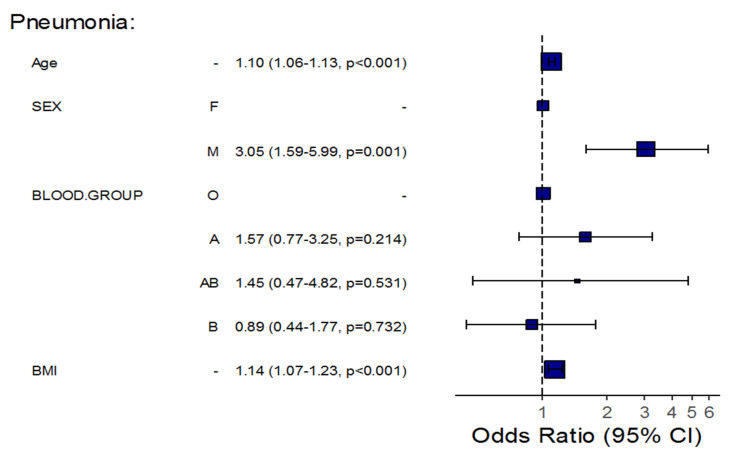
Regression Analysis of the Association between Blood Groups and Pneumonia, Taking into Consideration Patient Demographics.

**Figure 2:**
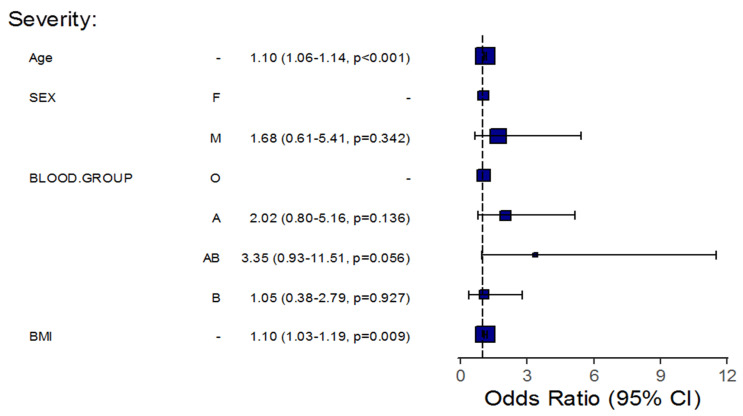
Regression Analysis of the Association between Blood Groups and Disease Severity, Taking into Consideration Patient Demographics.

**Figure 3:**
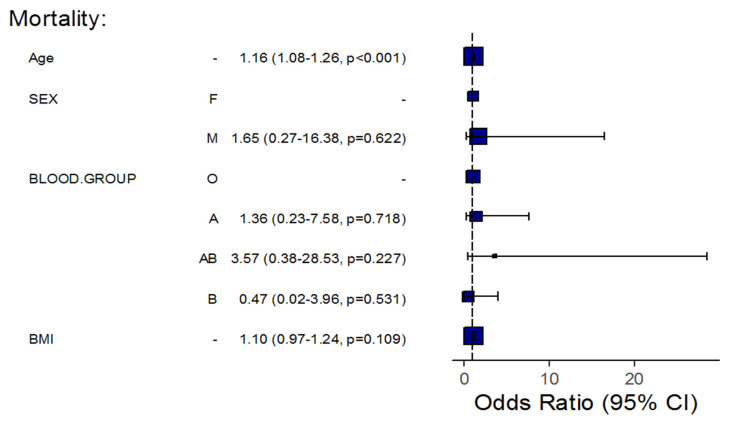
Regression Analysis of the Association between Blood Groups and Mortality, Taking into Consideration Patient Demographics.

Among COVID-19 patients with blood group B, the age-related risk for pneumonia and mortality was lower than that of patients with blood group O; each one-year increase in age demonstrated a lower risk for the odds of pneumonia and mortality, with an approximately 9% (OR = 0.911, *p* = 0.0179) and 25% (OR = 0.748, *p* = 0.045) decrease, respectively. Moreover, each one-unit increase in BMI demonstrated a significantly lower risk for the odds of pneumonia, with an approximately 20% decrease among patients of the same blood group (OR = 0.797, *p* = 0.0071) if compared to group O (Table 4, Figure 1, Figure 2 and Figure 3).

### 3.2. The Association between ABO Blood Groups and Laboratory Findings

A comparison between all blood groups in terms of different laboratory findings are presented in (Table S1). The linear regression models did not show any significant association between laboratory findings and blood groups, assuming that blood group O is exposed compared to the remaining non-O blood groups (Table 5).

There was a statistically significant increase in platelet count and troponin I, with a 58.51 (slope = 58.51, 95%CI: 10.19 to 106.82, *p* = 0.018) and 0.45 (slope = 0.45, 95%CI: 0.01 to 0.89, *p* = 0.046) increase, respectively, among patients with blood group A, assuming that blood group A is the exposed one compared to the remaining non-A blood groups (Table 6).

### 3.3. The Association between ABO Blood Groups and Laboratory Findings

**Figure 4:**
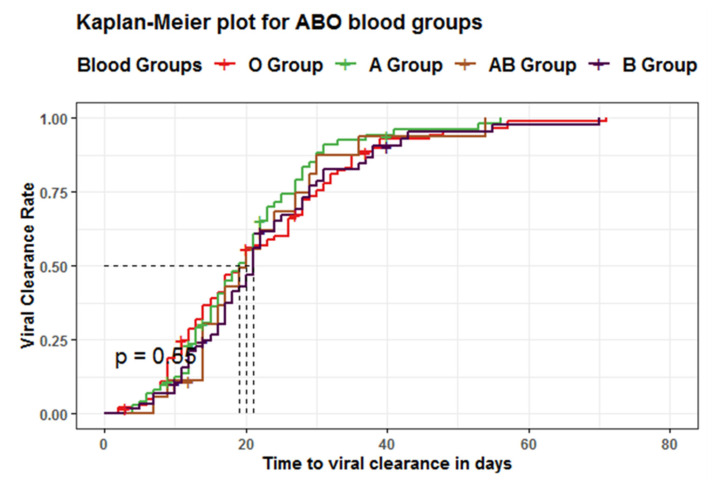
Kaplan–Meier Estimator in Relation to Blood Groups Using Log-Rank Test.

The Kaplan–Meier estimator was adjusted for censoring, including cases transferred to another hospital before viral clearance, some outpatients who had been lost to follow up, and deceased patients. The analysis showed a non-statistically significant difference between all blood groups regarding the median time to reach viral clearance using the log-rank test (*p* = 0.6, log-rank = 2.1) (Table 7 and Figure 4).

The COX proportional hazard model showed a non-significant association between patients’ blood groups and viral clearance rate. Furthermore, after adjusting for the RH group, it was found that patients with blood group B and a positive RH factor had a statistically significant shorter time to viral clearance; about four-fold when compared to patients with blood group O (RR = 4.15, 95%CI: 1.089–15.85, *p* = 0.037) (Table 8).

## 4. Discussion

COVID-19 has spread worldwide, with several risk factors for severe disease outcomes [17]. Health experts had suspected that ABO blood groups may impact rates of SARS-CoV-2 infection and disease outcomes. This study showed that blood group O was the most common among all patients, followed by blood groups A, B, and AB. RH positivity was prevalent in all patients; this could be explained by knowing that RH positivity is common within the population. In our study, we did not measure the association between RH positivity and outcomes of COVID-19. However, we found that COVID-19 patients of blood group B with RH positivity had a shorter time until viral clearance than other blood groups. Still, recent reports observed the protective effect of RH negativity against susceptibility to infection, intubation, and mortality among COVID-19 patients [12,18]; this inconsistency highlights the need to explore the impact of RH positivity among COVID-19 patients.

Scientists have hypothesized that the presence of any coexisting co-morbidity increases the risk of severe clinical outcome of COVID-19, which may lead to death. In our study, co-morbidities in COVID-19 patients included hypertension, diabetes mellitus, and cardiovascular problems; none of these co-morbidities were common among all included patients or inpatients. This could be attributed to the change of hospitalization criteria over time; the tentative period of this study was early in 2020, and at this time all SARS-CoV-2-positive cases were recommended to be hospitalized due to a lack of knowledge. Later, the hospitalization criteria were modified to include only severe and non-severe patients with co-morbidities which increased the risk of severe disease.

Wang et al. conducted a meta-analysis of 1558 COVID-19 patients with different co-morbidities; they reported that diabetes mellitus, cerebrovascular disease, hypertension, and cardiovascular disease could be considered independent risk factors for severe COVID-19 outcomes. It is important to identify risk factors as early as possible to guide clinicians in the appropriate medical management of populations at high risk of severe outcomes of COVID-19 [19].

Our study could not find an association between the ABO blood group and specific outcomes of COVID-19, including pneumonia, ICU admission, severe disease, the need for oxygen, and death. Additionally, we did not find any significant difference in any of the analyzed outcomes, either when comparing patients with blood group O or blood group A. The only exception was that blood group AB had statistically significant higher odds of disease severity than other blood groups when blood group O was the exposed group in the analysis.

After adjustment for age, BMI, and gender, we found that an increased age increased the risk for pneumonia, severe disease, and mortality among patients of all blood groups. A higher BMI increased the risk for pneumonia and mortality among patients of all blood groups. Additionally, being of male gender increased the risk for pneumonia among patients of all blood groups.

When COVID-19 patients of blood group B were compared to COVID-19 patients of blood group O, older age was linked to a lower risk of pneumonia and mortality, and a higher BMI was also linked to a lower risk of pneumonia. These findings emphasize the importance of a number of factors that influence the link between ABO blood types and COVID-19 etiology.

Similar to our findings, Latz et al. reported no association between ABO blood groups and ICU admission or discharge [20]. Furthermore, Hoiland et al. showed no association between mortality due to COVID-19 and ABO blood groups [21].

Inconsistently with our results, Zietz and his colleagues observed a decreased risk of intubation and mortality among COVID-19 patients of blood group A when compared with those of blood groups AB or O. However, those of blood group B were more exposed to intubation while less exposed to mortality in comparison with those of blood group O [18]. Latz et al. found that patients of blood group A were more protected against the severity and mortality of COVID-19 [20].

While Acik and Bankir had reported that patients of blood group O were less susceptible to pneumonia caused by SARS-CoV-2, the authors stated that a higher number of O blood group patients were in the included subjects; this could be a very reasonable interpretation [22].

Pourali and his colleagues found that blood group A was considered a risk factor while blood group O was a protective factor against SARS-CoV-2 infection. However, there was no association between all blood groups and death [11]. Samra et al. also reported a positive association between blood group A and mortality due to SARS-CoV-2 infection [17].

Based on the above observations, it is postulated that the increased risk for severe outcomes among patients with blood group A could be attributed to the increased frequency of thromboembolic disorders among individuals compared to those with blood group O. Additionally, plasma levels of VWF are lower by 25% in patients with blood group O compared to those with non-O blood groups, explaining the lower risk of thrombosis among those with blood group O. However, this postulation is still not strong enough to allow particular prophylaxis measurements among patients of blood groups other than O group [22,23].

Several mechanisms could explain the controversial results of other studies. Firstly, increased genetic susceptibility for respiratory failure caused by SARS-CoV-2 in the presence of the 3p21.31 gene cluster [14]. Secondly, the inhibitory effect of the human anti-A antibody on the adhesion between viral S protein and ACE2 among non-A blood group patients. Thirdly, the presence of A/B phenotypic-determining enzymes in individuals with blood groups A, B, or AB enhances the virus’s molecular contact. However, these enzymes are absent in those with blood group O [24]. The distribution of ABO blood types varies throughout populations, and the complexity of distinct characteristics impacting COVID-19 outcome could influence these results.

The association between ABO blood groups and SARS-CoV-2 infection could also be explained by the presence of anti-A and/or anti-B antibodies that serve as viral neutralizing antibodies by binding to A and/or B antigens expressed on the viral envelope, thereby preventing infection of target cells. The association can also be attributed to the inhibitory effect of the binding between SARS-CoV-2 S protein and anti-A antibodies on the interaction between the virus and ACE-2 receptor, thereby preventing entry into the lung epithelium. In addition, increased activity of ACE-1 in patients with blood group A predisposes to cardiovascular complications, responsible for severe COVID-19. Additionally, the variation of VWF and Factor VIII levels by ABO group with higher levels in these individuals contributes to risk of thromboembolic disease and severe COVID-19. Another mechanism may involve ABH glycans that could modify the affinity of SARS-CoV-2 for ACE-2 receptor, they could also serve as alternative, lower-affinity receptors for SARS-CoV-2 S protein or bind other viral envelope structures, affecting initial infection and disease severity [25].

We wanted to determine whether the ABO blood group is associated with clinical indicators, including laboratory parameters of COVID-19 patients. So, we compared all COVID-19 laboratory findings to ABO blood groups; we found a significant increase in creatinine among patients with blood group AB compared to other blood groups. We also found no significant difference between these lab results and the type of ABO blood group except in troponin and platelets among patients with blood group A, assuming that blood group A is exposed compared to the remaining non-A blood groups. There was no significant association between laboratory findings and blood groups, considering that blood group O is exposed compared to the remaining non-O blood groups. Later, we compared the time to viral clearance between different blood groups and found no statistically significant difference. However, adjustment for the RH groups resulted in a significant decrease in time till viral clearance among patients with blood group B.

Hoiland et al. have investigated laboratory parameters of COVID-19 in relation to ABO blood type. They observed significantly increased levels of several inflammatory markers and cytokines among severe and ICU admitted COVID-19 patients with blood groups A or AB compared to those with blood groups O or B. However, the difference in serum levels of IL-1β was not significant among all blood groups [21].

Our study’s limitations include the small sample size, along with the conducting of the study in the multiethnic community of the UAE, noting that blood group distribution varies by ethnicity, making precise risk comparison impossible. Additionally, our study was conducted early during the first wave of the pandemic, so patients infected with the wild type of SARS-CoV-2 were included.

Furthermore, we did not rule out or control for some confounding characteristics, such as age, ethnicity, or gender. Finally, our study only included hospitalized COVID-19 cases in the comparison analysis, with no healthy subjects.

## 5. Conclusions

In conclusion, we reported no significant association between the ABO blood group and outcomes of COVID-19, with the exception of blood group AB. There was a variable association between different ABO groups and COVID-19 laboratory findings among COVID-19 patients after adjustment for different demographic and clinical characteristics. Our findings and other reports in the literature highlight the impact of the ABO blood group and the pathogenesis of COVID-19. Still, the exact mechanism and the magnitude of the effect had significant differences that require further exploration through larger and more highly controlled comparative studies.

## Figures and Tables

**Table 1:** Comparative Analysis for Baseline Demographics, Co-Morbidities and COVID-19 Outcomes between All Blood Groups.

N = 303 (100%)		“O Group” 122 (40.3%)	“A Group” 84 (27.7%)	“AB Group” 21 (6.9%)	“B Group” 76 (25.1%)	*p* Value
Demographics						
Age (years)	Mean (SD)	38.6 (11.3)	38.4 (10.3)	41.9 (10.8)	40.7 (9.9)	0.174
BMI (kg/m^2^)	Mean (SD)	27.4 (5.0)	26.6 (5.7)	26.8 (3.3)	27.2 (3.8)	0.712
Sex	Female	39 (32.0%)	26 (31.0%)	4 (19.0%)	13 (17.1%)	0.085
	Male	83 (68.0%)	58 (69.0%)	17 (81.0%)	63 (82.9%)	
Race/Ethnicity	African	1 (0.8%)	0 (0.0%)	0 (0.0%)	2 (2.6%)	0.251
	Asian	106 (86.9%)	70 (83.3%)	18 (85.7%)	70 (92.1%)	
	Black	1 (0.8%)	1 (1.2%)	0 (0.0%)	1 (1.3%)	
	White	14 (11.5%)	13 (15.5%)	3 (14.3%)	3 (3.9%)	
RH Factor	Negative	18 (16.1%)	5 (6.5%)	1 (4.8%)	4 (5.8%)	0.077
	Positive	94 (83.9%)	72 (93.5%)	20 (95.2%)	65 (94.2%)	
Co-morbidities						
Hypertension	No	109 (91.6%)	68 (94.4%)	19 (95.0%)	59 (89.4%)	0.749
	Yes	10 (8.4%)	4 (5.6%)	1 (5.0%)	7 (10.6%)	
Diabetes Mellitus	No	110 (94.0%)	63 (85.1%)	19 (90.5%)	59 (89.4%)	0.222
	Yes	7 (6.0%)	11 (14.9%)	2 (9.5%)	7 (10.6%)	
Cardiovascular Diseases	No	118 (98.3%)	80 (97.6%)	20 (100.0%)	71 (94.7%)	0.43
	Yes	2 (1.7%)	2 (2.4%)	0 (0.0%)	4 (5.3%)	
COVID-19 Outcomes						
Radiology	Normal	60 (49.2%)	35 (41.7%)	7 (33.3%)	34 (44.7%)	0.495
	Pneumonia	62 (50.8%)	49 (58.3%)	14 (66.7%)	42 (55.3%)	
Icu Admission	No	116 (95.1%)	78 (92.9%)	18 (85.7%)	71 (93.4%)	0.418
	Yes	6 (4.9%)	6 (7.1%)	3 (14.3%)	5 (6.6%)	
Severity	No	108 (88.5%)	69 (82.1%)	15 (71.4%)	67 (88.2%)	0.152
	Yes	14 (11.5%)	15 (17.9%)	6 (28.6%)	9 (11.8%)	
Oxygen Need	No	114 (93.4%)	73 (86.9%)	17 (81.0%)	72 (94.7%)	0.079
	Yes	8 (6.6%)	11 (13.1%)	4 (19.0%)	4 (5.3%)	
Multi-Organ Failure/Renal Replacement Therapy	No	118 (96.7%)	83 (98.8%)	19 (90.5%)	75 (98.7%)	0.172
	Yes	4 (3.3%)	1 (1.2%)	2 (9.5%)	1 (1.3%)	
Mortality	No	117 (95.9%)	81 (96.4%)	19 (90.5%)	75 (98.7%)	0.279
	Yes	5 (4.1%)	3 (3.6%)	2 (9.5%)	1 (1.3%)	

**Table 2:** Logistic Regression Models Investigating the Association between Blood Groups and COVID-19 Outcomes (Making “O Group” the Reference Group).

	“A Group”		“AB Group”		“B Group”	
	OR (95%CI)	*p* Value	OR (95%CI)	*p* Value	OR (95%CI)	*p* Value
Pneumonia	1.35 (0.78–2.38)	0.288	1.94 (0.75–5.41)	0.184	1.20 (0.67–2.13)	0.543
ICU admission	1.49 (0.45–4.92)	0.505	3.22 (0.64–13.42)	0.119	1.36 (0.38–4.68)	0.621
Severity	1.68 (0.76–3.73)	0.199	3.09 (0.97–9.05)	0.044	1.04 (0.41–2.50)	0.938
Oxygen need	2.15 (0.83–5.79)	0.118	3.35 (0.82–11.93)	0.069	0.79 (0.21–2.61)	0.711
Multiorgan Failure, Renal Replacement Therapy	0.36 (0.02–2.46)	0.359	3.11 (0.41–17.09)	0.208	0.39 (0.02–2.72)	0.408
Mortality	0.87 (0.17–3.63)	0.848	2.46 (0.34–12.37)	0.301	0.31 (0.02–1.98)	0.292

**Table 3:** Logistic Regression Models Investigating the Association between Blood Groups and COVID-19 Outcomes (Making “A Group” the Reference Group).

	“O Group”		“AB Group”		“B Group”	
	OR (95%CI)	*p* Value	OR (95%CI)	*p* Value	OR (95%CI)	*p* Value
Pneumonia	0.74 (0.42–1.29)	0.288	1.43 (0.54–4.11)	0.487	0.88 (0.47–1.65)	0.695
ICU admission	0.67 (0.20–2.22)	0.505	2.17 (0.43–9.07)	0.305	0.92 (0.25–3.17)	0.888
Severity	0.60 (0.27–1.32)	0.199	1.84 (0.58–5.39)	0.277	0.62 (0.24–1.49)	0.290
Oxygen need	0.47 (0.17–1.20)	0.118	1.56 (0.40–5.22)	0.488	0.37 (0.10–1.13)	0.100
Multiorgan Failure, Renal Replacement Therapy	2.81 (0.41–55.57)	0.359	8.74 (0.80–193.84)	0.083	1.11 (0.04–28.31)	0.943
Mortality	1.15 (0.28–5.75)	0.848	2.84 (0.36–18.32)	0.270	0.36 (0.02–2.88)	0.381

**Table 4:** Multivariate Logistic Regression Models Investigating the Association between Blood Groups and COVID-19 Outcomes, Taking into Consideration Patient Demographics.

	Pneumonia				Severity				Mortality			
	Crude OR (95% CI)	*p*	Adj. OR (95% CI)	*p*	Crude OR (95% CI)	*p*	Adj. OR (95% CI)	*p*	Crude OR (95% CI)	*p*	Adj. OR (95% CI)	*p*
Age	1.12 (1.09–1.16)	**<0.001**	1.10 (1.06–1.13)	**<0.001**	1.10 (1.07–1.14)	**<0.001**	1.10 (1.06–1.14)	**<0.001**	1.16 (1.09–1.25)	**<0.001**	1.16 (1.08–1.26)	**<0.001**
BMI	1.18 (1.11–1.26)	**<0.001**	1.14 (1.07–1.23)	**<0.001**	1.12 (1.05–1.20)	**<0.001**	1.10 (1.03–1.19)	**0.009**	1.12 (1.01–1.23)	**0.017**	1.10 (0.97–1.24)	0.109
Sex (Male)	4.43 (2.58–7.79)	**<0.001**	3.05 (1.59–5.99)	**0.001**	2.63 (1.14–7.15)	**0.036**	1.68 (0.61–5.41)	0.342	1.70 (0.43–11.30)	0.504	1.65 (0.27–16.38)	0.622
Group O	**Reference group**											
Group A	1.35 (0.78–2.38)	0.288	1.57 (0.77–3.25)	0.214	1.68 (0.76–3.73)	0.199	2.02 (0.80–5.16)	0.136	0.87 (0.17–3.63)	0.848	1.36 (0.23–7.58)	0.718
Group AB	1.94 (0.75–5.41)	0.184	1.45 (0.47–4.82)	0.531	3.09 (0.97–9.05)	**0.044**	3.35 (0.93–11.51)	0.056	2.46 (0.34–12.37)	0.301	3.57 (0.38–28.53)	0.227
Group B	1.20 (0.67–2.13)	0.543	0.89 (0.44–1.77)	0.732	1.04 (0.41–2.50)	0.938	1.05 (0.38–2.79)	0.927	0.31 (0.02–1.98)	0.292	0.47 (0.02–3.96)	0.531
Age in group B	-	-	0.911	**0.0179**	-	-	-	-	-	-	0.748	**0.045**
BMI in group B	-	-	0.797	**0.0071**	-	-	-	-	-	-	-	-

**Table 5:** Linear Regression Models Investigating the Association between Blood Groups and COVID-19 Laboratory Findings among Inpatients (Making “O Group” the Exposed Group).

Laboratory Findings	Coefficient (95% CI)	*p*-Value
White Blood Cell Count (×10^9^/L)	0.44 (−0.61 to 1.49)	0.407
Hemoglobin (g/L)	−0.14 (−1.22 to 0.94)	0.799
Platelets (×10^9^/L)	−1.64 (−47.26 to 43.97)	0.943
C-Reactive Protein (mg/L)	−16.56 (−45.89 to 12.77)	0.266
D-dIMER (µg/mL)	−0.87 (−3.12 to 1.39)	0.449
Lactate Dehydrogenase (U/L)	−50.46 (−183.67 to 82.76)	0.455
Alanine Aminotransferase (U/L)	−2339.66 (−8075.41 to 3396.08)	0.421
Aspartate Aminotransferase (U/L)	−9.31 (−24.77 to 6.15)	0.236
Creatinine (mg/dL)	0.15 (−0.04 to 0.33)	0.127
Neutrophil Count (%)	−0.73 (−5.72 to 4.25)	0.771
Lymphocyte Count (%)	0.11 (−4.07 to 4.28)	0.960
Neutrophil to Lymphocyte Ratio	0.21 (−1.58 to 2.00)	0.817
Red Cell Distribution Width CV (%)	−0.14 (−0.84 to 0.56)	0.689
Fibrinogen (mg/dL)	−42.07 (−116.96 to 32.81)	0.269
Ferritin (ng/mL)	−37.00 (−493.66 to 419.65)	0.873
Prothrombin Time (sec)	−0.06 (−0.66 to 0.54)	0.840
International Normalized Ratio	−0.02 (−0.07 to 0.04)	0.536
Troponin I (ng/mL)	−0.20 (−0.62 to 0.22)	0.351
Procalcitonin (ng/mL)	0.18 (−0.11 to 0.47)	0.229
Glucose Mmol/L)	−1.69 (−3.91 to 0.52)	0.132

**Table 6:** Linear Regression Models Investigating the Association between Blood Groups and COVID-19 Laboratory Findings among Inpatients (Making “A Group” the Exposed Group).

Laboratory Findings	Coefficient (95% CI)	*p*-Value
White Blood Cell Count (×10^9^/L)	0.13 (−1.00 to 1.27)	0.819
Hemoglobin (g/L)	−0.83 (−1.98 to 0.33)	0.159
Platelets (×10^9^/L)	58.51 (10.19 to 106.82)	**0.018**
C-Reactive Protein (mg/L)	26.95 (−4.52 to 58.43)	0.093
D-dIMER (µg/mL)	1.54 (−0.88 to 3.95)	0.211
Lactate Dehydrogenase (U/L)	81.66 (−61.89 to 225.22)	0.263
Alanine Aminotransferase (U/L)	−1983.20 (−8189.67 to 4223.27)	0.529
Aspartate Aminotransferase (U/L)	1.43 (−15.48 to 18.34)	0.867
Creatinine (mg/dL)	−0.13 (−0.34 to 0.07)	0.201
Neutrophil Count (%)	1.90 (−3.48 to 7.28)	0.486
Lymphocyte Count (%)	−1.20 (−5.71 to 3.30)	0.599
Neutrophil to Lymphocyte Ratio	−0.27 (−2.20 to 1.67)	0.786
Red Cell Distribution Width CV (%)	−0.02 (−0.78 to 0.74)	0.961
Fibrinogen (mg/dL)	54.61 (−26.06 to 135.28)	0.183
Ferritin (ng/mL)	41.22 (−450.05 to 532.48)	0.869
Prothrombin Time (sec)	0.50 (−0.14 to 1.14)	0.121
International Normalized Ratio	0.02 (−0.04 to 0.07)	0.588
Troponin I (ng/mL)	0.45 (0.01 to 0.89)	**0.046**
Procalcitonin (ng/mL)	−0.05 (−0.36 to 0.25)	0.722
Glucose (Mmol/L)	1.72 (−0.80 to 4.25)	0.178

**Table 7:** Time to Viral Clearance in Relation to Blood Groups.

Blood Groups	Total Number of Events	Median time to Viral Clearance (Days)	95%CI (Days)	*p*-Value	Log-Rank
Group O	97	19	17–26	0.6	2.1
Group A	68	19	16–22		
Group AB	16	20	16–30		
Group B	53	21	18–24		

**Table 8:** Multivariate Cox Proportional Hazard Models Investigating the Association between Viral Clearance Rate (Rate Ratio) and Blood Groups.

		Crude HR (95% CI)	*p* Value	Adj. HR (95% CI)	*p* Value
Blood Group	O	Reference group			
	A	1.21 (0.89–1.66)	0.230	1.37 (0.45–4.15)	0.577
	AB	1.07 (0.63–1.82)	0.808	1.16 (0.67–1.997)	0.6012
	B	0.96 (0.68–1.34)	0.792	0.272 (0.076–0.968)	**0.0445**
RH	Negative	Reference group			
	Positive	1.14 (0.73–1.79)	0.553	0.806 (0.459–1.41)	0.4517
Positive RH: Group B		-	-	4.15 (1.089–15.85)	**0.037**

## Data Availability

Data is available upon request from the first and corresponding author.

## Supplementary Materials

The following supporting information can be downloaded at: https://www.mdpi.com/article/10.3390/life12081157/s1, Table S1: Comparative Analysis for Laboratory Findings between Blood Groups among Inpatients.
