# Supplementary material for: ABO Blood Group in Relation to COVID-19 Susceptibility and Clinical Outcomes: A Retrospective Observational Study in the United Arab Emirates
Source: Life (Basel). 2022 Jul 29;12(8):1157. doi: 10.3390/life12081157 (PMC9410437; doi:10.3390/life12081157)
Supplement: Supplementary file 1 [file life-12-01157-s001.zip › life-1791514-supplementary.pdf]

Supplementary Table S1: Comparative Analysis for Laboratory Findings between Blood Groups among Inpatients

Laboratory findings		"A group"	"AB group"	"B group"	"O group"	P value
White Blood Cells Count ($\times 10^9$/L)	Median (IQR)	7.0 (3.4)	6.6 (2.9)	6.4 (2.0)	6.7 (2.6)	0.9403
Hemoglobin (g/L)	Median (IQR)	13.2 (2.0)	13.8 (1.4)	14.5 (2.3)	14.2 (2.8)	0.1564
Platelets ($\times 10^9$/L)	Median (IQR)	304.0 (215.0)	233.5 (73.5)	287.0 (121.0)	271.0 (131.5)	0.05483
C-Reactive Protein (mg/L)	Median (IQR)	55.0 (105.0)	44.5 (78.8)	12.5 (60.8)	14.0 (68.2)	0.1064
D-dIMER (µg/mL)	Median (IQR)	0.6 (1.8)	0.4 (0.5)	0.3 (0.7)	0.5 (0.8)	0.2781
Lactate Dehydrogenase (U/L)	Median (IQR)	299.0 (294.0)	297.0 (274.0)	260.0 (207.0)	227.0 (148.8)	0.1435
Alanine Aminotransferase (U/L)	Median (IQR)	33.0 (24.7)	45.0 (21.8)	35.0 (40.0)	41.0 (37.5)	0.5312
Aspartate Aminotransferase (U/L)	Median (IQR)	39.0 (31.0)	55.0 (46.0)	37.0 (30.0)	36.0 (19.5)	0.4645
Creatinine (mg/dL)	Median (IQR)	0.8 (0.3)	1.1 (0.2)	0.8 (0.3)	0.9 (0.3)	0.01998
Neutrophil.Count (%)	Median (IQR)	64.6 (29.9)	61.5 (16.0)	57.2 (20.1)	59.8 (19.1)	0.896
Lymphocyte.Count (%)	Median (IQR)	24.8 (22.5)	27.9 (15.6)	31.7 (17.7)	28.6 (17.3)	0.8619
Neutrophil to Lymphocyte Ratio	Median (IQR)	2.5 (3.7)	2.2 (3.0)	1.6 (2.3)	2.1 (2.3)	0.8518
Red Cell Distribution Width.CV (%)	Median (IQR)	12.9 (1.0)	13.8 (1.3)	12.8 (1.0)	13.1 (1.0)	0.247
Fibrinogen (mg/dL)	Median (IQR)	628.0 (420.0)	494.0 (259.8)	513.5 (274.0)	467.0 (264.2)	0.5123
Ferritin (ng/mL)	Median (IQR)	365.0 (875.5)	473.5 (1809.6)	430.7 (556.8)	273.4 (652.0)	0.4573
Prothrombin Time (sec)	Median (IQR)	14.0 (2.0)	13.0 (0.5)	14.0 (1.0)	13.8 (1.6)	0.2592
International Normalized Ratio	Median (IQR)	1.0 (0.1)	1.0 (0.1)	1.0 (0.2)	1.0 (0.1)	0.4733
Troponin.I (ng/ml)	Median (IQR)	0.0 (0.0)	0.0 (0.0)	0.0 (0.0)	0.0 (0.0)	0.4212
Procalcitonin (ng/mL)	Median (IQR)	0.1 (0.2)	0.1 (0.3)	0.1 (0.0)	0.1 (0.1)	0.8754
Glucose (Mmol/L)	Median (IQR)	6.0 (3.5)	8.9 (8.0)	5.8 (1.5)	5.4 (3.0)	0.5764
